# Post-Hatch Performance of Broilers Following Hypoxic Exposure During Incubation Under Suboptimal Environmental Temperature

**DOI:** 10.3389/fphys.2022.934676

**Published:** 2022-07-22

**Authors:** A. Haron, D. Shinder, M. Ruzal, S. Druyan

**Affiliations:** ^1^ The Robert H. Smith Faculty of Agriculture, Food and Environment, Hebrew University of Jerusalem, Rehovot, Israel; ^2^ Institute of Animal Science, Agricultural Research Organization, Volcani Center, Rishon Le Ziyyon, Israel

**Keywords:** embryonic development, hypoxia, incubation, broiler performance, thermoregulation, metabolism

## Abstract

The modern broiler is selected to exploit its full genetic potential, to sustain a rapid growth rate, and to lower the feed conversion rate (FCR). Recently reported reductions in FCR have been associated with augmented tissue formation at the expense of physiological functions such as thermoregulation. In turn, modern broilers exhibit a relatively low capability to balance energy expenditure under suboptimal ambient temperature. Hypoxic conditions at late incubation stages play a role in reforming metabolic plasticity. This work examined the effect of exposure to 12-h hypoxia (12H; 17% O_2_) for three consecutive days (from E16 through E18), or continuous hypoxia exposure for 48 h (48H), from E16 through E17, as compared to standard incubation (21% O_2_) on post-hatch performance of broilers maintained under suboptimal ambient temperatures (cold, hot, and diurnal cyclic ambient temperature). 12H chicks kept under hot ambient temperature had significantly lower body temperature (Tb) as compared to the control chicks. On day 42, both 12H and 48H chicks grown in the cyclic temperature room had significantly lower Tbs than controls. In parallel, from week 4, onward, 12H chicks had a significantly lower FCR than controls, and the 48H chicks demonstrated a lower FCR from week 5 and on. 12H and 48H broilers maintained under diurnal cyclic ambient temperature, exhibited significantly greater relative breast muscle weight, and a similar pattern was found in hypoxic broilers raised under standard and hot ambient temperatures. Hypoxic manipulation affects and create an adaptive bias in allocating metabolic energy between maintenance and growth, thus resulting in improved broiler performance, thermoregulation, and rearing under suboptimal environmental temperature.

## 1 Introduction

Since the 1950s, commercial genetic selection programs have led to dramatic improvements in broiler production traits. Genetic selection for performance traits resulted in considerable enhancement in daily feed consumption, elevated metabolic rate (Tickle et al., 2018), and consequently elevated internal heat production. These, in turn, translated to rapid growth, high feed conversion efficiency, and higher meat production characteristics (Havenstein et al., 1994; Zuidhof et al., 2014). While highly desirable for efficient production traits, such developments logically necessitate parallel increases in the size of the cardiovascular and respiratory systems, as well as enhancements in their functional efficiency (Druyan 2008). However, insufficient development of these critical systems has led to a relatively low capability to adequately maintain dynamic steady-state mechanisms in the body, that should balance energy expenditure and body water balance under suboptimal environmental conditions (Yahav, 2009). The phenomenon is highlighted by the limited broiler energy budget (Tickle et al., 2018), which provides minimal allowance for increasing resting metabolic rate (RMR) to support the energetic resources available for growth when thermoregulatory costs are increased. All this leads to insufficient maintenance of the dynamic steady-state of thermoregulation processes, resulting in the enhancement of body temperature (Tb) fluctuations.

Broilers have developed certain responses in order to cope with environmental stress. The direct responses stimulated by environmental conditions have been characterized as acclimation/acclimatization (Yahav et al., 1997; Horowitz, 1998). While, adaptive, maladaptive or neutral with regard to an individual’s fitness (Ghalambor et al., 2007) ability of a phenotype to be modified by the environment (Bradshaw, 1965), is characterized as a phenotypic plasticity. Phenotypic plasticity may involve short-term, reversible changes within an individual or developmental plasticity, which involves irreversible changes that result from developmental processes (Piersma and Drent, 2003). More specifically, plasticity underlies changes in age-dependent susceptibility of an embryo or juvenile animal to environmental stressors, due to changes in the development of their physiological regulatory systems (Spicer and Burggren, 2003). Environmental changes during critical developmental time windows, e.g., structural and/or functional shaping of the control of physiological/neurological systems, can disrupt and alter the developmental trajectory. However, the same condition outside of the critical window has little or no effect, and, in some cases, may have a negative effect (Carroll, 2003; Spicer and Burggren, 2003).

Hypoxia during broiler embryo incubation has been found to trigger adaptation of the embryonic cardiovascular system to the altered environment, with elevations in blood parameters, such as hematocrit, hemoglobin (Haron et al., 2017), and heart rate (Tomi et al., 2019). The actual effects of hypoxia on embryo development depend on the critical period of exposure, hypoxia level, and duration of hypoxic exposure. Hypoxia during embryonic development was reported to affect growth and metabolism of embryos, contingent on the hypoxia regimen (Haron et al., 2022). Amaral-Silva et al. (2017) showed that 15% O_2_ during the last third of the incubation led to lower hatchling body mass. Haron et al. (2022) reported that while exposure for 48 h to 17% O_2_ during the plateau period was associated with lower body weight at hatch, embryos subjected to cycling exposure of 12 h–21/17% O_2_ between E16 and E18, had similar body weights at hatch to those of chicks incubated under standard conditions. The intermediate cycling hypoxia protocol enables embryos to adequately adapt to the shortage of oxygen and compensate for the gap that developed after the first exposure window before hatching (Haron et al., 2017, 2022). Moderate hypoxic exposure during the plateau period seems to trigger metabolic adaptation. The combination of lower Tb at hatch and lower plasma thyroid hormone concentrations in hypoxic manipulate hatchlings suggest that metabolic plasticity (Haron et al., 2017, 2022) and/or decreased heat production (Piestun et al., 2008a; Collin et al., 2011) underlie the responses to hypoxic exposure.

This study evaluated the effects of exposure of broiler embryos to a moderate O_2_ concentration (17%) during the plateau phase of embryonic development on, growth rate and feed conversion of broilers maintained under suboptimal environmental (ambient temperature) conditions up to the age of poultry marketing.

## 2 Materials and Methods

### 2.1 Experimental Design

#### 2.1.1 Egg Origin and Incubation

Cobb (500) strain broiler chicken eggs (*n* = 900) with an average weight of 62.0 ± 2.5 g, were obtained from a breeder flock of hens during their optimal period of egg production (35 weeks old). Eggs were individually numbered and weighed and then incubated in a 2,500-egg incubator (Danki ApS, Ikast, Denmark) under standard incubation conditions of 37.8°C and 56% relative humidity (RH), with turning once per hour (ended on E18). The incubator was located 31 m above sea level, with 20.9% O_2_ in the air. At E16_0 after candling, fertile eggs were randomly assigned to one of three treatment groups (300 eggs per treatment):

1) O_2_ concentration of 17% for 12 h per day (h/d) from E16 through E18 (designated as 12H). The eggs were exposed at three time point: E16_0 to E16_12, E17_0 to E17_12, and E18_0 to E18_12.
2) Continuous exposure to 17% O_2_, from E16 through E17, a total of 48 h (designated as 48H).
3) Control—O_2_ concentration of 21%.

Exposure to 17% O_2_ was accomplished by transferring eggs from both hypoxia treatment groups to an incubator with 17% O_2_ equipped with a Model 2BGA-SP-MA O_2_ and CO_2_ Control System (Emproco Ltd., Ashkelon, Israel) the incubation conditions of 37.8°C and 56% relative humidity (RH), with turning once per hour were kept. The O_2_ sensor activated an electronically controlled pump that infused N_2_ into the incubator to maintain the oxygen concentration at 17% ± 0.2%, while the CO_2_ level was 0.03 ± 0.01, as previously described by (Druyan et al., 2012).

On E19, all eggs from all treatment groups were transferred to hatching trays. In all three incubation treatments hatching stood on 95%.

#### 2.1.2 Rearing and Growing Period

At hatch, 80 male chicks from each incubation treatment were selected, individually tagged and weighed. Chicks from each group were divided into groups of 10, and raised together in battery cages until the age of 14 days. The birds were maintained under the recommended temperature regime according to broiler management guide (https://www.cobb-vantress.com/assets/Cobb-Files/045bdc8f45/Broiler-Guide-2021-min.pdf). At the age of 14 days, 20 chicks from each treatment group, were subjected to one of four thermal condition, in one of four controlled-environment rooms):

1) Cold ambient temperature, starting from 27°C on day 14 with a reduction of 1°C a day to constant 16°C at day 23 onwards.
2) Standard ambient temperature (control), starting from 27°C on day 14 with a reduction of 0.5°C a day to a constant 24°C at day 19 onwards.
3) Hot ambient temperature, starting from 27°C on day 14 with an increase of 0.7°C a day to constant 32°C at day 21 onwards.
4) Diurnal cyclic ambient temperature, starting from 27°C on day 14, with an increase of 0.7°C a day to constant 32°C at day 21 onwards during daytime (12 h). During night time, from 27°C on day 14 with a reduction of 0.5°C a day to a constant 24°C at day 19 onwards. Leading to environmental temperature of 32°C daytime and 24°C nighttime from day 21 onwards.

In all four environmental conditions, RH was 55% and light:dark cycles of 20:4 h were implemented. Water and feed in mash form were available for *ad libitum* consumption, with diet designed to meet the breeder recommendation for broilers. The diet consisted of a “pre-starter” (d 0–10d), “starter” (11–21d), grower (21–28d), and finisher (28d to marketing 42d) feed, with respective contents of crude protein (%) and energy (cal/kg ME) of: 22 and 3,035, 21.5 and 3,100, 20 and 3,180, and 19 and 3,250.

The chicks were distributed in the rooms randomly, with one chick per cage. Each chick was weighed every week, and weekly food consumption was calculated for each individual chick; FCR (kg of feed consumed/kg of live body weight) was calculated for each chick in each treatment. At the end of the experiment, at the age of 42 day, the chickens were individually weighed, and the feed was removed 12 h before slaughter. Breast muscle, abdominal fat pad, heart, and liver were removed and weighed and their weights were calculated relative to their live body weight.

### 2.2 Measurements

#### 2.2.1 Body Weight

Chicks were weighed on a weekly basis using a Sartorius Signum SIWADCP-V14 scale (capacity ± readability 35 kg ± 1 g). Weight gain was calculated as the difference between the current body weight of the chick and the body weight at the previous measurement.

#### 2.2.2 Feed Intake

Feed was weighed on a weekly basis, using a Sartorius Signum SIWADCP-V14 scale. Feed consumption per chick was calculated by subtraction of out feeder weight at the end of the week from the in feeder weight at the beginning of the week in order to calculate chick’s weekly feed consumption.

#### 2.2.3 Feed Conversion Rate

FCR was calculated by dividing each chick’s feed consumption by its weight gain during the period (per week or for the entire growth phase).

#### 2.2.4 Body Temperature Measurements

Broiler Tb was measured at weekly intervals using a digital thermometer (Super Speed Digital Thermometer; Procare Measure Technology Co., San Chung City, Taipei, Taiwan) with ±0.1°C accuracy, that was inserted 1.5 cm into the cloaca. Temperature was measured for 10 chicks per treatment.

#### 2.2.5 Organ Weight Following Slaughter

At 42 d of age, chickens were individually weighed and the feed was removed for 12 h prior to slaughter. Breast muscle, abdominal fat pad heart, and liver were removed and weighed, and their relative weights calculated based on live body weight.

### 2.3 Statistical Methods

Due to the high ascites mortality and morbidity, performance of broilers from all three incubation treatments kept under cold temperature was negatively affected, with less than 10 birds per incubation treatment. Data of broilers kept under cold temperature was amiss from the statistical analysis (only standard, hot, and cycling effects were tested).

Individual growth performance, feed consumption, FCR, and slaughter data were statistically processed using two-way ANOVA, according to the model:

Y=µ+ treatment+temp+treatment Xtemp+e

with treatment (Control, 12H and 48H) and ambient temperature (standard, hot, and cycling) as the main fixed effects, and their interaction (treatment × temperature).

No significant interactions were found (treatment × temperature) the data is given in Supplementary Tables S1–S5.

In order to study how hypoxic incubation advantageous broilers performance under each sub-optimal condition, individual growth performance, feed consumption, FCR, and slaughter data were statistically processed within each ambient temperature using one-way ANOVA, according to the model:

Y=µ+ treatment+e

with treatment (Control, 12H and 48H) as the main fixed effect.

Values that differed (at a level of *p* ≤ 0.05) were considered statistically significant. In addition, the Tukey test was conducted to compare the averages of the treatment effect.

### 2.4 Ethics Approval

All the procedures in this study were carried out in accordance with the accepted ethical and welfare standards of the Israel Ethics Committee (IL-581/15).

## 3 Results

### 3.1 Mortality During the Rearing Period

Mortality during rearing did not exceed 5% in groups exposed to control, hot or diurnal cyclic thermal condition in all three incubation treatments. In contrast, under cold thermal conditions, mortality was significantly higher (pχ2 ≤ 0.001) than in the other three groups, mainly due to manifestation of ascites syndrome, with 48%, 45%, and 39% mortality in the control, 12H and 48H chickens, respectively. Total percentage of ascites was 69%, 55%, and 50% in the control, 12H and 48H chickens, respectively. Due to the high ascites mortality and morbidity, data of broilers kept under cold temperature was amiss from the performances statistical analysis. No significant differences in mortality or ascites rates were observed across incubation treatments subgroups within each of the four thermal condition groups.

### 3.2 Body Weight and Growth During the Rearing Period

Table 1 shows the mean body weights and Table 2 shows the mean daily growth rate per week (end of each week) of chickens from different incubation treatments during their rearing under standard, hot or diurnal cyclic ambient temperature (starting from 14 days of age).

**TABLE 1:** Body weight (g) of broilers exposed to different hypoxia regimes during embryonic development and then raised under different environmental ambient temperatures (analyzed within each ambient temperature).

Age (d)	Ambient temperature								
	Standard—23°C			Hot—32°C			Diurnal cyclic—24°C–32°C		
	Con	12H	48H	Con	12H	48H	Con	12H	48H
0	44.0 ± 0.4	44.2 ± 0.4	44.5 ± 0.4	43.8 ± 0.4	44.2 ± 0.4	43.5 ± 0.4	44.3 ± 0.4	44.5 ± 0.4	44.4 ± 0.4
7	203.7 ± 2.8	200.5 ± 2.9	198.9 ± 3.0	197.6 ± 2.7	198.3 ± 2.8	199.0 ± 2.9	200.9 ± 2.7	199.3 ± 2.7	205.0 ± 2.8
14	554.0 ± 7.5	552.9 ± 7.5	550.4 ± 7.9	556.3 ± 7.3	556.8 ± 7.5	552.0 ± 7.7	556.4 ± 7.3	553.2 ± 7.3	551.2 ± 7.5
21	1039.6 ± 15.3	1038.7 ± 15.7	1044.1 ± 16.7	1050.0 ± 14.9	1046.1 ± 15.3	1030.7 ± 15.7	1089.3 ± 14.9	1075.0 ± 14.9	1061.8 ± 15.3
28	1753.8 ± 28.2	1749.5 ± 28.2	1726.3 ± 29.9	1505.3 ± 26.8	1567.2 ± 27.3	1556.9 ± 28.2	1743.5 ± 26.8	1742.1 ± 27.5	1743.1 ± 27.5
35	2609.1 ± 48.9	2535.6 ± 50.2	2547.7 ± 55.3	1961.72 ± 46.3	2037.9 ± 48.8	2038.3 ± 50.2	2383.8 ± 46.3	2474.4 ± 48.8	2455.7 ± 48.8
42	3283.9 ± 62.5	3285.0 ± 70.9	3355.0 ± 76.6	2221.1 ± 59.3	2318.8 ± 62.5	2364.5 ± 64.4	2833.5^*^ ± 59.3	3049.3^*^ ± 66.3	3009.9^*^ ± 62.5

Means ± SE are presented. *n* = 20 for each incubation treatment group under each environmental condition.

* On each day, Different letters indicate significant differences (*p* ≤ 0.05) across incubation treatments within each ambient temperature.

**TABLE 2:** Weight gain (g/d) of broilers that were exposed to different hypoxia regimes during embryonic development and then raised under different environmental ambient temperatures (analyzed within each ambient temperature).

Age (d)	Ambient temperature								
	Standard—23°C			Hot—32°C			Diurnal cyclic—24°C–32°C		
	Con	12H	48H	Con	12H	48H	Con	12H	48H
7	22.8 ± 0.4	22.3 ± 0.4	22.1 ± 0.4	22.0 ± 0.4	22.0 ± 0.4	22.1 ± 0.4	22.4 ± 0.4	22.1 ± 0.4	22.9 ± 0.5
14	50.0 ± 0.8	50.4 ± 0.8	50.3 ± 0.9	51.2 ± 0.9	51.2 ± 0.9	51.0 ± 0.8	50.8 ± 0.8	50.6 ± 0.8	49.5 ± 0.8
21	69.4 ± 1.6	69.4 ± 1.6	70.5 ± 1.7	70.5 ± 1.9	69.9 ± 1.9	70.5 ± 1.9	76.1 ± 1.5	74.5 ± 1.5	72.9 ± 1.6
28	100.5 ± 3.1	101.5 ± 3.1	97.5 ± 3.3	65.0^*^ ± 2.9	74.4^*^ ± 3.0	72.6^*^ ± 3.0	93.5 ± 2.9	95.7 ± 3.0	97.3 ± 3.0
35	112.2 ± 4.6	111.1 ± 4.7	113.3 ± 5.2	65.2 ± 4.4	67.8 ± 4.6	67.9 ± 4.7	91.5^*^ ± 4.4	104.4^*^ ± 4.6	103.3^*^ ± 4.6
42	96.3 ± 4.7	99.7 ± 4.7	100.2 ± 5.7	37.0 ± 4.4	40.1 ± 4.7	46.6 ± 4.8	64.2^*^ ± 4.4	83.5^*^ ± 5.0	79.2^*^ ± 4.7

Means ± SE are presented. *n* = 20 for each incubation treatment group under each environmental condition.

* On each day, different letters indicate significant differences (*p* ≤ 0.05) across incubation treatments within each ambient temperature.

The main effect on broiler body weights (Supplementary Table S1) and growth rate (Supplementary Table S2) was the thermal condition; from the age of 21d onward, there were significant differences in the two parameters between the rearing conditions groups. The weights of birds from all three incubation group were significantly heavier when reared under standard ambient temperature, while birds that were maintained under a hot ambient temperature had a significantly lower body weight than expected on day 42 (Supplementary Table S1). Significant effect of incubation condition on broilers body weight and growth was found at the last week of growth at day 42, with higher body weight and growth rate of the hypoxic incubated chicks as compare to control (Supplementary Tables S1, S2).

In general, body weights and growth of all broilers from the three incubation treatments within each rearing subgroups was similar, regardless of the ambient temperature conditions. The exception, was found at day 42 for broilers raised under Hot and Diurnal cyclic temperature condition. In both ambient conditions, chicks exposed to either the 12H or 48H schedule of hypoxia during incubation had higher body weights as compared to the control group 42d broilers. This difference in body weight was significant for broiler kept under Diurnal cyclic temperature condition (3049.3 ± 62.6 and 3009.9 ± 59.1 vs. 2833.5 ± 56.0 g for the 12H, 48H and control birds, respectively) (Table 1). As with body weight, ambient temperature had the most significant effect on broiler growth (Supplementary Table S2). During the entire period, within all environmental conditions, weight gain of the broilers was similar, except for broilers maintained under diurnal cyclic temperature, where 35d and 42d broilers from both hypoxia treatment groups had significantly higher growth compared to the control group broilers (Table 2), similar but non-significant pattern was found under standard and hot ambient temperature.

### 3.3 Body Temperature During the Rearing Period

When all birds were raised under standard brooding conditions (hatch to 14 days), Tb of chickens from the three incubation treatment groups was similar. Exposure to different ambient temperatures thereafter, affected chicken Tb, with a marked increase in Tb among chickens maintained under hot environmental conditions (35°C) (Supplementary Table S3). From day 21 onward, significant effect was also found to incubation conditions, with standard broilers Tb higher to significantly higher from 12H to 48H broilers Tb. Within each of the environments, the pattern of difference in Tb between standard to hypoxic broilers was maintained (Table 3). While under standard ambient temperature, the differences between standard and hypoxic broiler Tb wasn’t constant, under suboptimal ambient conditions, Tb of 12H and 48H chickens was lower throughout the growth period compared to control. This difference in Tb across incubation groups was significant between 21-day-old 12H and control broilers maintained under a hot ambient temperature (41.6°C ± 0.1°C, 41.7°C ± 0.1°C, and 41.8°C ± 0.1°C for the 12H, 48H, and control broilers, respectively). On Day 42 in the diurnal cyclic ambient temperature, both hypoxia-treated groups demonstrated significantly lower Tb compared the control (41.3 ± 0.1 and 41.2°C ± 0.1°C vs. 41.5°C ± 0.1°C for the 12H, 48H, and control embryos, respectively; Table 3).

**TABLE 3:** Body temperature (°C) of broilers exposed to different hypoxia regimes during embryonic development and then raised under different environmental ambient temperatures (analyzed within each ambient temperature).

Age (d)	Ambient temperature								
	Standard—23°C			Hot—32°C			Diurnal cyclic—24°C–32°C		
	Con	12H	48H	Con	12H	48H	Con	12H	48H
0	40.5 ± 0.1	40.5 ± 0.1	40.5 ± 0.1	40.6 ± 0.1	40.5 ± 0.1	40.5 ± 0.1	40.6 ± 0.1	40.4 ± 0.1	40.1 ± 0.1
7	41.4 ± 0.1	41.3 ± 0.1	41.3 ± 0.1	41.3 ± 0.1	41.2 ± 0.1	41.4 ± 0.1	41.4 ± 0.1	41.3 ± 0.1	41.3 ± 0.1
14	41.4 ± 0.1	41.3 ± 0.1	41.4 ± 0.1	41.4 ± 0.1	41.3 ± 0.1	41.3 ± 0.1	41.3 ± 0.1	41.2 ± 0.1	41.3 ± 0.1
21	41.3 ± 0.1	41.3 ± 0.1	41.2 ± 0.1	41.8^*^ ± 0.1	41.6^*^ ± 0.1	41.7^*^ ± 0.1	41.5 ± 0.1	41.4 ± 0.1	41.4 ± 0.1
28	41.5 ± 0.1	41.4 ± 0.1	41.4 ± 0.1	42.4 ± 0.1	42.2 ± 0.1	42.2 ± 0.1	41.6 ± 0.1	41.5 ± 0.1	41.6 ± 0.1
35	41.5 ± 0.1	41.5 ± 0.1	41.5 ± 0.1	42.6 ± 0.1	42.4 ± 0.1	42.4 ± 0.1	41.8 ± 0.1	41.6 ± 0.1	41.7 ± 0.1
42	41.5 ± 0.1	41.4 ± 0.1	41.5 ± 0.1	42.5 ± 0.1	42.3 ± 0.1	42.4 ± 0.1	41.5^*^ ± 0.1	41.3^*^ ± 0.1	41.2^*^ ± 0.01

Means ± SE are presented. *n* = 20 for each incubation treatment group under each environmental condition.

* On each day, different letters indicate significant differences (*p* ≤ 0.05) across incubation treatments within each ambient temperature.

### 3.4 Feed Intake and Feed Conversion Rate During Exposure to Different Ambient Temperatures

The different ambient temperatures had a significant effect on broiler feed consumption, with the highest consumption during the entire brooding period (14–42 days) measured under standard ambient temperature and the lowest under hot condition (Supplementary Table S4). Despite the absence of a significant difference in feed intake among incubation treatment groups grown post-hatch under different environments, there was a trend of lower feed intake among 12H broilers as compared to control broilers when reared under standard, or diurnal cyclic ambient temperatures (Table 4).

**TABLE 4:** Feed intake (kg per week) of broilers exposed to different hypoxia regimes during embryonic development and then raised under different environmental ambient temperatures (analyzed within each ambient temperature).

Age (wk)	Ambient temperature								
	Standard—23°C			Hot—32°C			Diurnal cyclic—24°C–32°C		
	Con	12H	48H	Con	12H	48H	Con	12H	48H
3 weeks	720 ± 16.6	723 ± 17.6	702 ± 18.1	687 ± 16.2	685 ± 17.1	678 ± 17.1	769 ± 16.2	765 ± 17.1	737 ± 17.1
4 weeks	1132 ± 24.3	1109 ± 27.5	1175 ± 27.5	941 ± 24.3	967 ± 24.3	1001 ± 24.3	1100^*^ ±23.6	1038^*^ ± 24.3	1078^*^ ± 23.6
5 weeks	1444 ± 52.3	1418 ± 59.3	1463 ± 64.1	963 ± 49.6	994 ± 50.9	977 ± 52.3	1236 ± 50.9	1216 ± 53.8	1299 ± 50.9
6 weeks	1406 ± 43.1	1354 ± 48.8	1425 ± 52.7	829 ± 43.1	784 ± 45.7	811 ± 45.7	1256 ± 45.7	1277 ± 48.8	1295 ± 44.3
Total:3–6 weeks	4709 ± 94.6	4599 ± 111.3	4871 ± 121.0	3499 ± 100.3	3493 ± 107.3	3542 ± 100.3	4401 ± 100.3	4388 ± 111.3	4387 ± 100.3

Means ± SE are presented. *n* = 20 for each treatment incubation group under each environmental condition.

* On each day, different letters indicate significant differences (*p* ≤ 0.05) across incubation treatments within each ambient temperature.

Succeeding the effect of ambient temperatures on food intake and growth, a significant effect was also found on FCR of the broilers (Supplementary Table S5).

From week 4 and onward, the slight difference in growth combined with the trend in feed intake of broilers from the different incubation treatments, resulted in significant lower FCR of 12H broiler as compare to control with 48H broilers rank in the middle (1.80 ± 0.02 vs. 1.81 ± 0.02 and 1.85 ± 0.02 for the 12H, 48H, and control broilers, respectively; Supplementary Table S5).

A comparison between incubation treatments within each ambient temperature found that while the difference in FCR was found between groups of broilers in all three ambient temperature, it was highly significant when the broilers were raised under diurnal cyclic ambient temperature.

Under diurnal cyclic ambient temperature, significant lower FCR (Table 5) was found for both hypoxic broilers groups as compared to the control from week 4 onward. The total FCR (14–42d) of the hypoxia-exposed broilers was significantly lower and more efficient compared to that of the control broilers (1.73 ± 0.03 vs. 1.78 ± 0.02 and 1.85 ± 0.02 for the 12H, 48H, and control broilers, respectively).

**TABLE 5:** Feed-conversion ratios (FCR) of broilers that were exposed to different hypoxia regimes during embryonic development and then raised under different environmental ambient temperatures (analyzed within each ambient temperature).

Age (week)	Ambient temperature								
	Standard—23°C			Hot—32°C			Diurnal cyclic—24°C–32°C		
	Con	12H	48H	Con	12H	48H	Con	12H	48H
3 weeks	1.49 ± 0.02	1.49 ± 0.02	1.45 ± 0.03	1.40 ± 0.02	1.38 ± 0.02	1.38 ± 0.02	1.45 ± 0.02	1.46 ± 0.02	1.43 ± 0.02
4 weeks	1.62 ± 0.04	1.55 ± 0.04	1.61 ± 0.05	1.96^*^ ± 0.04	1.81^*^ ± 0.05	1.98^*^ ± 0.04	1.64^*^ ± 0.04	1.55^*^ ± 0.03	1.58^*^ ± 0.03
5 weeks	1.70 ± 0.09	1.74 ± 0.10	1.70 ± 0.11	2.33 ± 0.08	2.27 ± 0.09	2.22 ± 0.09	1.97^*^ ± 0.08	1.78^*^ ± 0.04	1.84^*^ ± 0.04
6 weeks	2.11 ± 0.20	1.94 ± 0.22	2.05 ± 0.24	3.54 ± 0.20	2.69 ± 0.21	2.62 ± 0.21	2.49^*^ ± 0.08	2.10^*^ ± 0.08	2.24^*^ ± 0.07
Total: 3–6 weeks	1.73 ± 0.03	1.70 ± 0.03	1.72 ± 0.04	1.98 ± 0.03	1.96 ± 0.03	1.93 ± 0.03	1.86^*^ ± 0.03	1.73^*^ ± 0.03	1.78^*^ ^,b^ ± 0.02

Means ± SE are presented. *n* = 20 for each treatment incubation group under each environmental condition.

* On each day, different letters indicate significant differences (*p* ≤ 0.05) across incubation treatments within each ambient temperature.

### 3.5 Organ Weight Following Slaughter

**FIGURE 1:**
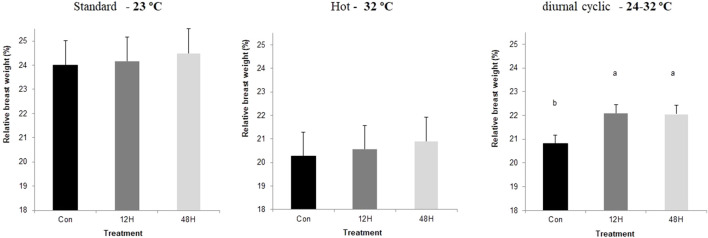
Relative breast weight (%final body weight at 42 day), of broilers that were exposed to different hypoxia regimes (12H and 48H) during embryonic development and then raised under different environmental ambient temperatures (cold, standard, hot, and diurnal cyclic ambient temperature).

**FIGURE 2:**
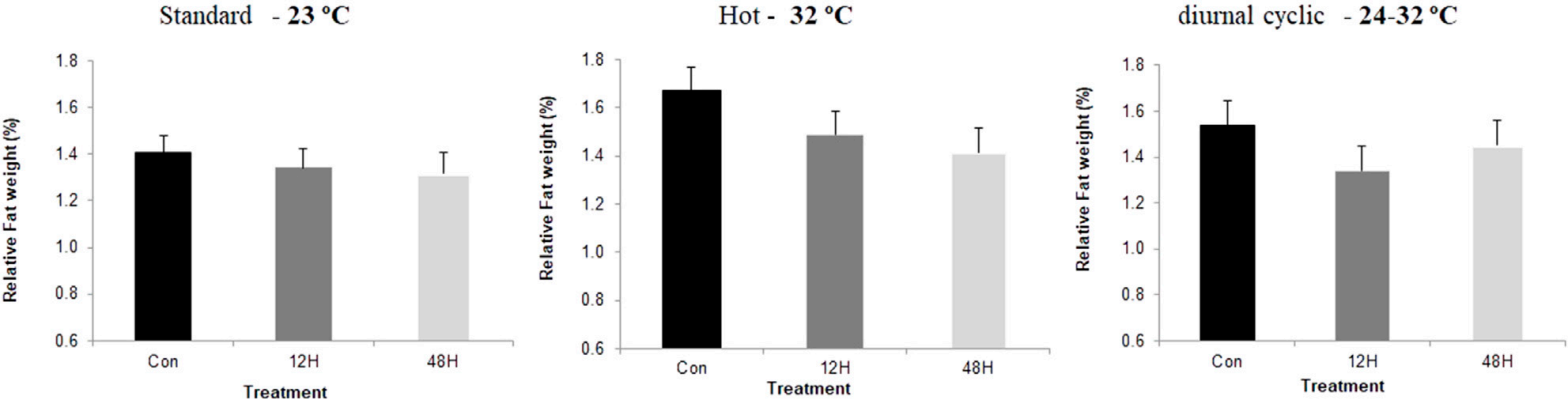
Relative abdominal fat pad (%final body weight at 42 day), of broilers that were exposed to different hypoxia regimes (12H and 48H) during embryonic development and then raised under different environmental ambient temperatures (cold, standard, hot, and diurnal cyclic ambient temperature).

Slaughter took place on day 43, after 12 h without feeding. Environmental ambient temperature significantly affected broilers performance with a significant reduction in meat yield and breast relative weight, when broilers from all incubation treatments were kept under suboptimal ambient temperatures, with broilers kept under hot ambient temperature exhibiting the lowest relative breast weight (Figure 1). Despite the absence of a significant difference in relative breast weight among incubation treatment groups, there was a trend of a higher relative breast weight among broilers of both hypoxia-incubated groups later exposed to standard and hot ambient temperatures. This trend was significant in hypoxic incubated broilers maintained under diurnal cyclic ambient temperature. Under hot ambient temperature, relative abdominal fat pad was heavier, as expected, with broilers from both hypoxic incubation treatments exhibiting lower relative abdominal fat weight compared to control broilers. A similar trend was also found under standard and diurnal cyclic ambient temperatures as well (Figure 2). No difference in relative heart weight or relative liver weight was found between broilers from all three-incubation group under the different ambient conditions.

## 4 Discussion

Developmental changes induced by environmental conditions during incubation may impact post-hatch growth and metabolism (Shinder et al., 2007; Yahav and Brake, 2014). During chicken embryonic development, several critical windows associated with sensitivity to hypoxia (Druyan et al., 2012; Haron et al., 2017; Ben-Gigi et al., 2021), control of ventilation (Ferner and Mortola, 2009), thermal manipulation (Piestun et al., 2009a, 2011), and metabolic rate (Tazawa et al., 2004) have been defined. Hypoxia has been demonstrated an environmental incubation condition that can affect embryonic development, cardiovascular development, metabolism, O_2_ demand, and the available energy for post-hatch growth and development (Haron et al., 2021). The latter is based on the assumption that environmental factors have a strong influence on determination of the “set-point” for physiological control systems during “critical developmental phases” (Dörner, 1974; Shinder et al., 2011).

Recent studies by Haron et al. (2017, 2021, 2022) demonstrated that hypoxic exposure during the plateau stage led to alterations in metabolism and cardiovascular system of the developing embryo, which resulted in more efficient energy utilization. These prior studies concluded that moderate hypoxic exposure from E16 to E18 elicited a long-lasting effect on the energy-balance axis, which suggested a metabolic plasticity and/or decreased heat production (Haron et al., 2022). The combination of lower Tb at hatch and lower plasma thyroid hormone concentrations in the 12H broilers suggested a long-lasting effect of hypoxia on post-hatching thermotolerance (Haron et al., 2022).

This work examined the effect of exposure to 12-h hypoxia (17% O_2_) for three consecutive days (from E16 through E18) as compared to continuous hypoxia exposure for 48 h, from E16 to E17, on post-hatch performance of broilers maintained under suboptimal environmental temperatures (cold, hot, and diurnal cyclic ambient temperature). The technique of coping with sub-optimal conditions might emphasize advantageous traits of the hypoxic incubated broilers that are not evident under standard conditions.

While rearing the broiler under hot or diurnal cyclic ambient temperature caused redundant mortality, raising the broilers under cold ambient temperature led to ascites syndrome (Druyan, 2012). Total percentages of ascites were 69%, 55%, and 50% in the control, 12H and 48H chickens, respectively. Druyan et al. (2007, 2008) hypothesized that the tendency to develop the syndrome is associated with high growth rate, only because the latter increases oxygen demand in the genetically susceptible individuals above a threshold reflecting their lower inherent oxygen supply capacity. Druyan et al. (2012); Druyan and Levi, 2012) concluded that embryos adapted to hypoxic condition have an increased oxygen consumption capacity, which enables their growth, development, and maturation to proceed as well as those of control embryos. Although hypoxia exposure reduced the manifestation of ascites in the hypoxia treatment groups compared to control, the difference was not significant.

In this study, all chicks exhibited similar performance in the early stages of growth, regardless of their incubation treatments. The first significant difference between broilers from the three-incubation treatments was observed on day 21, 7 days after introduction to the new ambient temperature; 12H chicks had significantly lower Tb as compared to the control chicks raised in the hot room, similar pattern was also found under diurnal cyclic temperature. This difference in Tb signifying 12H broilers ability to maintain the Tb in the thermo-neutral zone, despite the hot environment. Amaral-Silva et al. (2017) reported that despite the fact that 10-day-old hypoxia-incubated chicks had higher oxygen consumption than controls, animals of both groups had a similar Tb. They suggested that hypoxic incubation may have also decreased heat conservation/increased heat loss, in comparison to control animals at this age.

Energy consumption, in general, and in the domestic fowl, in particular, is divided between maintenance and production. In endotherms, the ability to maintain Tb depends on daily energy requirement for maintenance. Therefore, lowering the demands for maintenance, while preserving an approximately constant total energy consumption, allows for increased allocation of energy toward production. An alternative beneficial metabolic response may consist of a combination of reduced maintenance energy demands coupled with an overall reduction in energy consumption. Intermittent thermal manipulation during broiler embryo development and maturation of the thyroid axis has been shown to have a long-lasting effect on energy balance of broilers subjected to hot environments, improving thermo-tolerance and performance parameters (Piestun et al., 2011).

Without an interaction between incubation conditions and ambient temperature, it appear that hypoxic incubation had affected broilers metabolism in terms of internal heat production (lower body temperature), energy utilization and in terms of FCR. Raising the hypoxic manipulated broilers under sub-optimal environmental conditions helped emphasize the long lasting effect it had on metabolic plasticity. From all tested environmental conditions it appears that, raising the broilers under diurnal cyclic ambient temperature exposed the most the effect of hypoxic incubation on broilers performance, whereas when raised under other ambient temperatures the pattern was present but no significant differences were found between treatments.

On the last day of the growth trial (day 42), broilers were at their peak body weight and with maximum energy demand in the tissues. By this time point, both 12H and 48H chicks grown in the cyclic temperature room had significantly lower Tbs than controls. In parallel, from week 4, 1 week after introduction to the cyclic temperature, until week 6, 12H chicks had a significantly lower FCR than controls, and the 48H chicks demonstrated a lower FCR from week 5 and onward. Taken together, despite their lower Tbs and lower feed intake, hypoxia-exposed chicks maintained their growth rate, while the control chicks failed to maintain a low Tb, indicating higher energy expenditure on maintenance.

Another effect that can be related to reduce requirements for maintenance energy was the relative size of the breast muscle, which was used in this study as a central indicator organ for growth and development. When broilers from both hypoxic groups were maintained under diurnal cyclic ambient temperature, they exhibited significantly greater relative breast muscle weight, and a similar pattern was found in broilers raised under standard and hot ambient temperatures. This increase was associated with reduced abdominal fat, suggesting better energy channeling towards growth rather than accumulation of fat (Piestun et al., 2013b, 2013a). Similar results were reported by Druyan et al. (2018), who suggested that hypoxia exposure during incubation has an angiogenic effect on breast muscle, likely leading to improved nutrient delivery to the breast, and to subsequent utilization of for tissue growth rather than for fat accumulation.

## 5 Conclusion

Hypoxic conditions during the incubation period may improve post-hatch chick growth, metabolism, and health. Mild hypoxia exposure during chick embryogenesis causes alterations in metabolism and cardiovascular system, improving chorioallantoic membrane (CAM) and cardiovascular development, with a subsequent improvement in O_2_-carrying capacity.

Once hypoxic conditions return to normal, development of exposed embryos not only progresses, but follows a unique developmental trajectory, demonstrating developmental plasticity that has an effect on post-hatch chick performance and enables improved adaptation to additional environmental stress, such as suboptimal environmental conditions. This outcome is of economic and practical relevance for the commercial broiler rearing phase, where a shift from the natural thermal zone to either the lower or upper critical level can compromise productivity.

## Data Availability

The raw data supporting the conclusion of this article will be made available by the authors, without undue reservation.
