# Supplementary material for: Post-Hatch Performance of Broilers Following Hypoxic Exposure During Incubation Under Suboptimal Environmental Temperature
Source: Front Physiol. 2022 Jul 22;13:934676. doi: 10.3389/fphys.2022.934676 (PMC9355520; doi:10.3389/fphys.2022.934676)
Supplement: Supplementary file 1 [file DataSheet1.docx]

**Table 1.** Body weight (g) of broilers exposed to different hypoxia regimes during embryonic development and then raised under different environmental ambient temperatures by main effects.

|  | Incubation | | | Ambient temperature | | | | Significance (Prob F)  of main and interaction effects^1.2^ | | |
| --- | --- | --- | --- | --- | --- | --- | --- | --- | --- | --- |
| **Age (d)** | **Con** | **12H** | **48H** | | **standard - 23 ºC** | **Hot - 32 ºC** | **diurnal cyclic - 24-32 ºC** | **Incubation2** | **Thermal 1** | **Incubation X thermal** |
| 0 | 44.0±0.2 | 44.3±0.2 | 44.1±0.3 | | 44.2±0.3 | 43.8±0.2 | 44.4±0.2 | ns | ns | ns |
| 7 | 200.7±1.6 | 199.3±1.6 | 200.8±1.7 | | 201.0±1.7 | 198.1±1.6 | 201.7±1.6 | ns | ns | ns |
| 14 | 555.6±4.2 | 554.3±4.3 | 552.2±4.2 | | 552.4±4.4 | 556.1±4.3 | 553.6±4.2 | ns | ns | ns |
| 21 | 1059.7±8.7 | 1053.3±8.8 | 1051.5±9.2 | | 1040.8±9.2^B^ | 1048.3±8.7^AB^ | 1075.4±8.7^A^ | ns | 0.0167 | ns |
| 28 | 1667.5±15.7 | 1686.2±16.0 | 1675.4±16.5 | | 1743.2±16.6^A^ | 1543.1±16.6^B^ | 1742.9±15.7^A^ | ns | <0.0001 | ns |
| 35 | 2318.3±27.2 | 2349.2±28.5 | 2347.2±29.8 | | 2564.2.3±29.8^A^ | 2012.6±28.0^C^ | 2438.0±27..7^B^ | ns | <0.0001 | ns |
| 42 | 2779.4±34.9^b^ | 2884.4±38.5^ab^ | 2909.8±39.3^a^ | | 3307.9±40.9^A^ | 2301.5±35.9^C^ | 2964.2±36.3^B^ | 0.0299 | <0.0001 | ns |
| Means±SE are presented. *n*=60 for each incubation group, with 60 chicks per environmental condition.  ^1^On each day, different, capital letters (A-C) indicate significant differences (p ≤ 0.05) among ambient thermal condition effects.  ^2^on each day Different letters indicate significant differences (p≤0.05) across incubation treatments. | | | | | | | | | | |

**Table 2.** Weight gain (g/d) of broilers that were exposed to different hypoxia regimes during embryonic development and then raised under different environmental ambient temperatures.

|  | Incubation | | | Ambient temperature | | | Significance (Prob F)  of main and interaction effects^1.2^ | | |
| --- | --- | --- | --- | --- | --- | --- | --- | --- | --- |
| **Age (d)** | **Con** | **12H** | **48H** | **standard - 23 ºC** | **Hot - 32 ºC** | **diurnal cyclic - 24-32 ºC** | **Incubation** | **Thermal** | **Incubation X thermal** |
| 7 | 22.4±0.2 | 22.1±0.2 | 22.4±0.2 | 22.4±0.2 | 22.0±0.2 | 22.5±0.2 | ns | ns | ns |
| 14 | 50.7±0.5 | 50.7±0.5 | 50.2±0.5 | 50.2±0.5 | 51.1±0.5 | 50.3±0.5 | ns | ns | ns |
| 21 | 72.0±0.9 | 71.3±0.9 | 71.3±0.9 | 69.8±0.9^B^ | 70.3±0.9^B^ | 74.5±0.9^A^ | ns | 0.0003 | ns |
| 28 | 86.3±1.7 | 90.6±1.7 | 89.1±1.8 | 99.8±1.8^A^ | 70.7±1.8^B^ | 95.5±1.7^A^ | ns | <0.0001 | 0.09 |
| 35 | 93.0±2.6 | 94.4±2.6 | 94.8±2.8 | 115.5±2.8^A^ | 67.0±2.6^C^ | 99.7±2.6^B^ | ns | <0.0001 | ns |
| 42 | 65.9±2.6^b^ | 74.4±2.9^a^ | 75.3±2.9^a^ | 98.7±3.0^A^ | 41.3±2.7^C^ | 75.7±2.7^B^ | 0.0273 | <0.0001 | ns |
| Means±SE are presented. *n*=60 for each incubation group, with 60 chicks per environmental condition.  ^1^On each day, different, capital letters (A-C) indicate significant differences (p ≤ 0.05) among ambient thermal condition effects.  ^2^on each day Different letters indicate significant differences (p≤0.05) across incubation treatments. | | | | | | | | | |

**Table 3.** Body temperature (ºC) of broilers exposed to different hypoxia regimes during embryonic development and then raised under different environmental ambient temperatures.

|  | Incubation | | | Ambient temperature | | | | Significance (Prob F)  of main and interaction effects^1.2^ | | |
| --- | --- | --- | --- | --- | --- | --- | --- | --- | --- | --- |
| **Age (d)** | **Con** | **12H** | **48H** | | **standard - 23 ºC** | **Hot - 32 ºC** | **diurnal cyclic - 24-32 ºC** | **Incubation** | **Thermal** | **Incubation X thermal** |
| 0 | 40.5±0.1 | 40.4±0.1 | 40.4±0.1 | | 40.5±0.1 | 40.5±0.1 | 40.4±0.1 | ns | ns | ns |
| 7 | 41.3±0.1 | 41.3±0.1 | 41.3±0.1 | | 41.3±0.1 | 41.3±0.1 | 41.3±0.1 | ns | ns | ns |
| 14 | 41.3±0.1 | 41.3±0.1 | 41.3±0.1 | | 41.3±0.1 | 41.3±0.1 | 41.3±0.1 | ns | ns | ns |
| 21 | 41.5±0.1^a^ | 41.4±0.1^b^ | 41.4±0.1^b^ | | 41.3±0.1^C^ | 41.7±0.1^A^ | 41.4±0.1^B^ | 0.0037 | <0.0001 | ns |
| 28 | 41.8±0.1^a^ | 41.8±0.1^ab^ | 41.7±0.1^n^ | | 41.5±0.1^C^ | 42.3±0.1^A^ | 41.6±0.1^B^ | 0.0233 | <0.0001 | ns |
| 35 | 42.0±0.1 | 41.9±0.1 | 41.9±0.1 | | 41.5±0.1^C^ | 42.5±0.1^A^ | 41.7±0.1^B^ | 0.08 | <0.0001 | ns |
| 42 | 41.9±0.1^a^ | 41.7±0.1^b^ | 41.7±0.1^b^ | | 41.5±0.1^B^ | 42.4±0.1^A^ | 41.3±0.1^B^ | 0.0192 | <0.0001 | ns |
| Means±SE are presented. *n*=60 for each incubation group, with 60 chicks per environmental condition.  ^1^On each day, different, capital letters (A-C) indicate significant differences (p ≤ 0.05) among ambient thermal condition effects.  ^2^on each day Different letters indicate significant differences (p≤0.05) across incubation treatments. | | | | | | | | | | |

**Table 4.** Feed intake (kg per week) of broilers exposed to different hypoxia regimes during embryonic development and then raised under different environmental ambient temperatures.

|  | Incubation | | | Ambient temperature | | | Significance (Prob F)  of main and interaction effects^1.2^ | | |
| --- | --- | --- | --- | --- | --- | --- | --- | --- | --- |
| **Age**  **(wk)** | **Con** | **12H** | **48H** | **standard - 23 ºC** | **Hot - 32 ºC** | **diurnal cyclic - 24-32 ºC** | **Incubation** | **Thermal** | **Incubation X thermal** |
| 3wk | 725±9.4 | 724±10.0 | 705±10.1 | 715±10.1^B^ | 683±9.7^C^ | 757±9.7^A^ | ns | <0.0001 | ns |
| 4wk | 1058±13.9 | 1038±14.7 | 1085±14.5 | 1139±15.3^A^ | 970±14.0^C^ | 1072±14.0^B^ | 0.0784 | <0.0001 | ns |
| 5wk | 1214±29.4 | 1209±31.6 | 1247±32.4 | 1442±33.9^A^ | 978±29.4^C^ | 1251±30.0^B^ | ns | <0.0001 | ns |
| 6wk | 1164±25.4 | 1138±27.6 | 1177±27.6 | 1395±27.9^A^ | 808±25.9^C^ | 1276±26.7^B^ | ns | <0.0001 | ns |
| Total:  3-6wk | 4203±56.8 | 4160±63.5 | 4267±62.2 | 4726±63.2^A^ | 3511±59.3^C^ | 4392±60.1^B^ | ns | <0.0001 | ns |
| Means±SE are presented. *n*=60 for each incubation group, with 60 chicks per environmental condition.  ^1^On each day, different, capital letters (A-C) indicate significant differences (p ≤ 0.05) among ambient thermal condition effects.  ^2^on each day Different letters indicate significant differences (p≤0.05) across incubation treatments. | | | | | | | | | |

**Table 5.** Feed-conversion ratios (FCR) of broilers that were exposed to different hypoxia regimes during embryonic development and then raised under different environmental ambient temperatures.

|  |  | | | Ambient temperature | | | Significance (Prob F)  of main and interaction effects^1.2^ | | |
| --- | --- | --- | --- | --- | --- | --- | --- | --- | --- |
| **Age**  **(wk)** | **Con** | **12H** | **48H** | **standard - 23 ºC** | **Hot - 32 ºC** | **diurnal cyclic - 24-32 ºC** | **Incubation2** | **Thermal** | **Incubation X thermal** |
| 3wk | 1.44±0.01 | 1.44±0.01 | 1.42±0.01 | 1.48±0.01 | 1.39±0.01 | 1.44±0.01 | ns | <0.0001 | ns |
| 4wk | 1.74±0.02^a^ | 1.63±0.02^b^ | 1.73±0.02^a^ | 1.59±0.02^B^ | 1.92±0.02^A^ | 1.59±0.02^B^ | 0.0022 | <0.0001 | ns |
| 5wk | 2.0±0.05 | 1.93±0.05 | 1.92±0.05 | 1.71±0.06^B^ | 2.27±0.05^A^ | 1.86±0.05^B^ | ns | <0.0001 | ns |
| 6wk | 2.71±0.12^a^ | 2.25±0.13^b^ | 2.30±0.13^b^ | 2.03±0.13^B^ | 2.95±0.12^A^ | 2.28±0.12^B^ | 0.0133 | <0.0001 | ns |
| Total:  3-6wk | 1.85±0.02^a^ | 1.80±0.02^b^ | 1.81±0.02^ab^ | 1.72±0.02^C^ | 1.96±0.02^A^ | 1.79±0.02^B^ | 0.0677 | <0.0001 | ns |
| Means±SE are presented. *n*=60 for each incubation group, with 60 chicks per environmental condition.  ^1^On each day, different, capital letters (A-C) indicate significant differences (p ≤ 0.05) among ambient thermal condition effects.  ^2^on each day Different letters indicate significant differences (p≤0.05) across incubation treatments. | | | | | | | | | |
